# Preparing to Grasp Emotionally Laden Stimuli

**DOI:** 10.1371/journal.pone.0045235

**Published:** 2012-09-14

**Authors:** Laura Alice Santos de Oliveira, Luís Aureliano Imbiriba, Maitê Mello Russo, Anaelli A. Nogueira-Campos, Erika de C. Rodrigues, Mirtes G. Pereira, Eliane Volchan, Cláudia Domingues Vargas

**Affiliations:** 1 Laboratório de Neurobiologia II, Programa de Neurobiologia, Instituto de Biofísica Carlos Chagas Filho, Universidade Federal do Rio de Janeiro, Rio de Janeiro, Brasil; 2 Programa de pós-graduação em ciências da reabilitação, Centro Universitário Augusto Motta (UNISUAM), Rio de Janeiro, Brasil; 3 Escola de Educação Física e Desportos, Universidade Federal do Rio de Janeiro, Rio de Janeiro, Brasil; 4 Instituto Biomédico, Universidade Federal Fluminense, Niterói, Brasil; University of Bologna, Italy

## Abstract

**Background:**

Contemporary theories of motor control propose that motor planning involves the prediction of the consequences of actions. These predictions include the associated costs as well as the rewarding nature of movements’ outcomes. Within the estimation of these costs and rewards would lie the valence, that is, the pleasantness or unpleasantness of a given stimulus with which one is about to interact. The aim of this study was to test if motor preparation encompasses valence.

**Methodology/Principal Findings:**

The readiness potential, an electrophysiological marker of motor preparation, was recorded before the grasping of pleasant, neutral and unpleasant stimuli. Items used were balanced in weight and placed inside transparent cylinders to prompt a similar grip among trials. Compared with neutral stimuli, the grasping of pleasant stimuli was preceded by a readiness potential of lower amplitude, whereas that of unpleasant stimuli was associated with a readiness potential of higher amplitude.

**Conclusions/Significance:**

We show for the first time that the sensorimotor cortex activity preceding the grasping of a stimulus is affected by its valence. Smaller readiness potential amplitudes found for pleasant stimuli could imply in the recruitment of pre-set motor repertoires, whereas higher amplitudes found for unpleasant stimuli would emerge from a discrepancy between the required action and their aversiveness. Our results indicate that the prediction of action outcomes encompasses an estimate of the valence of a stimulus with which one is about to interact.

## Introduction

Motor systems are exquisitely adapted to transform an action goal into a movement of greatest fit in a given context. This transformation is thought to be performed through internal models of actions, which operate by continuously monitoring the motor output and making future predictions of changes in body states and the immediate environment [1], [2]. Optimal control models have recently incorporated the concept of value [3], [4], defined herein as the costs as well as the rewarding outcomes of an action. Within this theoretical framework, if one makes a movement to achieve a rewarding state, the very nature of this reward should exert an influence on its planning and execution [3], [5]. The influence of reward on motor planning was inferred from kinematic studies [5], [6]. Likewise, motor planning might incorporate the costs of a given action.

A network of brain regions at the sensorimotor interface has been recurrently implicated in stimuli value encoding [3], [7]–[15]. In particular, cortical sensorimotor representations seem dynamically sensitive to value-laden circumstances [9], [11], [16]. For instance, recent mapping of primate parieto-frontal cortices with 500-ms trains of electrical pulses have revealed a collection of behaviorally relevant motor repertoires whose recruitment is triggered as if in accordance with a specific context [9], [17], [18]. This evidence suggested that motor preparation should be affected by the putative interaction with emotionally laden objects.

Studies in humans are consistent with the idea that emotional contexts engage motor circuits. For instance, it has already been shown that the processing of emotional stimuli modulates signals in motor-related areas and that a pivotal node of interaction between emotion and motor signals might be the midcingulate cortex [19]. This brain region has been proven to trigger a motivational drive for action, conveying affective information to other components of the motor circuits, including the primary motor cortex [19]–[21]. Likewise, experimentally induced emotional states have been reported to produce robust modulation of activity in primary motor and premotor cortices as well as in putamen [22]–[24].

Nevertheless, to the best of our knowledge, there is still no direct evidence whether the emotional valence of a stimulus with which one is about to interact influences motor planning. A means to capture this influence is through the measuring of the readiness potential. In 1965, Deecke and Kornhuber reported a pioneering study concerning the existence of electroencephalographic activity preceding a voluntary movement in humans [25]. The readiness potential has since been recognized as an electrophysiological marker of motor preparation [25]–[34] and has been classically described in two components [25]. While an early readiness potential component starts bilaterally around two seconds in the supplementary motor area (SMA), the late component begins mainly contralateral to the moving limb in the primary motor (M1) and pre-motor cortices at about 400 ms prior to the movement onset [35], [36]. Available experimental evidence suggests that the readiness potential magnitude is proportional to movement complexity, to force production, to the degree of effort involved in its execution [25], [37] and to sensorimotor resource mobilization [38]. Readiness potential magnitude is also inversely proportional to the easiness in performing a given movement [39].

Since Darwin’s seminal observations [40], emotions are considered as adaptive in the sense that they prompt actions that are beneficial to the organism. Thus, for any possible action, one should be able to predict the associated costs as well as the rewarding nature of its outcomes (value). Here we propose that within the estimation of these costs and rewards would lie the valence of stimuli with which one is about to interact. In other words, as actions often occur in emotionally laden contexts, emotional valence of the stimulus which one is about to grab should incorporate into the motor planning of that action. To investigate this assumption, we measured the readiness potential preceding the interaction with emotionally laden stimuli presented in transparent cylinders. We hypothesized that grasping and bringing pleasant stimuli toward the body might recruit preset approach-like motor repertoires. Moreover, the same action directed towards unpleasant stimuli might require overpassing preset networks to avoid them. Thus, the intrinsic costs associated to the interaction with unpleasant stimuli would be translated in larger readiness potentials whereas pleasant, potentially rewarding stimuli would be associated to smaller readiness potentials as compared to the interaction with neutral stimuli.

## Results

### Stimuli Selection

A set of thirty-nine^1^ stimuli consisting of thirteen pleasant, thirteen unpleasant and thirteen neutral items inside transparent cylinders was selected from an initial set of sixty stimuli (see Methods) based on the emotional categorization of the self-assessment manikin. Analyses of appropriateness of selection were conducted. Two repeated measures ANOVA, one for valence and another for arousal, were used to compare the respective ratings of the three stimuli categories. There was a main effect for valence [F (2, 24) = 288.84, p<0.001]. Fisher’s post-hoc comparisons indicated that valence ratings of pleasant stimuli (mean 7.4±0.62 standard deviation) were significantly higher and valence ratings of unpleasant stimuli (3.1±0.44) were significantly lower than those of neutral stimuli (5.0±0.21). There was also a main effect for arousal [F (2, 24) = 116.52, p<0.001]. Fisher’s post-hoc comparisons indicated that arousal ratings for pleasant and unpleasant stimuli (5.0±0.75 and 4.6±0.48, respectively) were each statistically different from neutral (1.8±0.27). No statistical difference was found between these two sets of ratings. Thus, pleasant and unpleasant stimuli provided equivalent arousal ratings. Importantly, the weight of the three sets of stimuli was not statistically different [F (2, 36) = 1.05, p = 0.36].

NOTE^1^: PLEASANT: Chocolate candy, chocolate tablet, money, wrapped condom, mobile phone, some soccer cards, two car toys, marbles, gold trophy, ball, television remote control, MP3 and wrist watch; UNPLEASANT: chicken gizzard, cake with hair, artificial vomit, embalmed cockroach, artificial excrement, embalmed rotten food, bluebottle fly on a biscuit, embalmed dead rat, rotten artichoke, embalmed chicken foot, artificial spider, artificial snake and embalmed fish eye; NEUTRAL: adhesive tape, pencil sharpener, crumpled paper ball, silver paper clips, binder clips, sponge, stick glue, piece of plastic bag, alkaline battery, cotton balls, pieces of colored wire, spun wool and strip of staples.

### Readiness Potential

**Figure 1:**
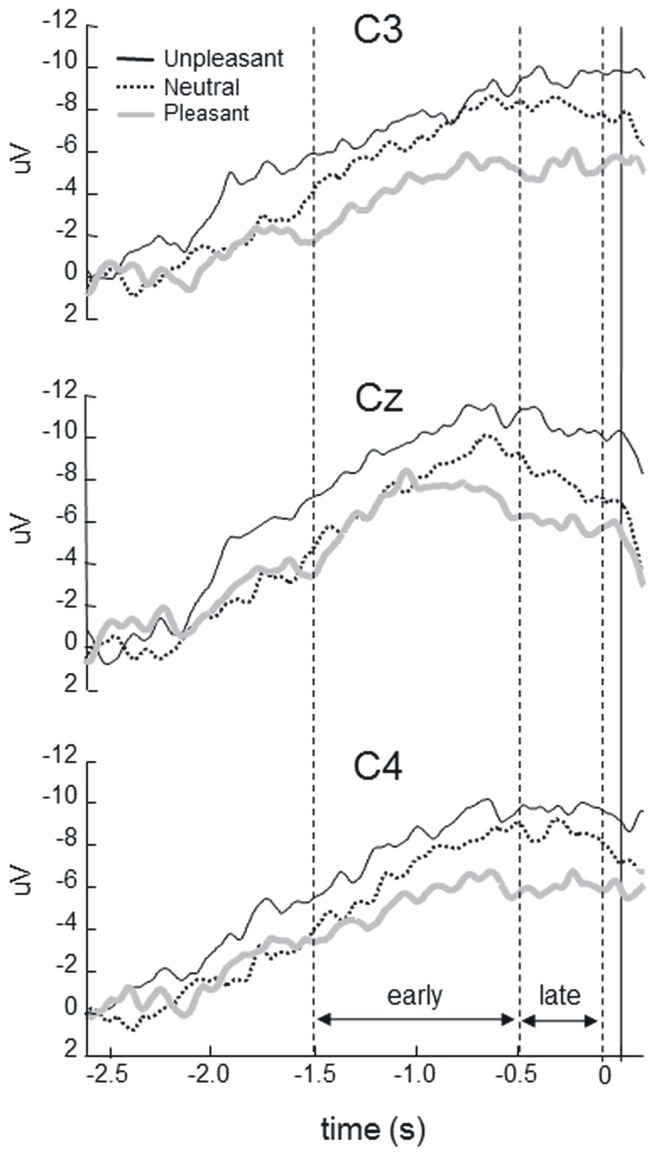
Mean average of readiness potential amplitudes preceding the grasping of pleasant, unpleasant and neutral stimuli for each analyzed channel (C3, CZ and C4). The mean readiness potential amplitude found for unpleasant stimulus was higher and that for pleasant stimuli lower relative to the observed for neutral stimuli.

Brain activity in the sensorimotor cortex preceding the grasping of pleasant, unpleasant and neutral stimuli was measured in eleven participants (Figure 1). Irrespective of the channel, the readiness potential for the unpleasant category appears as the outer contour while that for the pleasant category appears as the inner contour. A main effect was found for category [F (2, 20) = 12.06, p<0.01]. Fisher’s post-hoc analyses revealed that the mean readiness potential amplitude for the unpleasant category (mean −9.3±4.37 standard deviation) was significantly higher (p = 0.04) than that of the neutral category (−7.6±3.72). Furthermore, the mean readiness potential amplitude was significantly lower (p = 0.01) for the pleasant category (−5.6±3.7) than for the neutral category (Figure 1). Finally, the mean readiness potential amplitude was significantly higher (p<0.01) for the unpleasant category than for the pleasant category.

An interaction between channel and wave segment was found [F (2, 10) = 3.64, p<0.05]. Fisher’s post-hoc analyses revealed that, as expected, the late readiness potential mean amplitude was significantly higher than the early readiness potential mean amplitude both in C3 and C4 channels (p<0.01), see Table 1 for qualitative data. Also, mean amplitudes in C4 (early = −6.8; late = −8.2) were higher than in C3 (early = −6.1; late = −7.6) irrespective to the categories although this difference did not reach statistical significance.

**Table 1:** Mean and standard deviation values of readiness potential amplitudes.

Category	Wave segment	C3	CZ	C4
Unpleasant	Early	**−7.5 (3.09)**	−9.8 (4.79)	**−8.2 (3.42)**
	Late	**−9.67 (4.37)**	−10.7 (5.67)	**−9.8 (3.27)**
Pleasant	Early	**−4.3 (3.07)**	−6.8 (3.97)	**−5.3 (2.32)**
	Late	**−5.2 (4.17)**	−5.9 (4.41)	**−6.1 (3.05)**
Neutral	Early	**−6.6 (3.46)**	−7.7 (4.20)	**−6.8 (2.94)**
	Late	**−8.1 (3.56)**	−8.0 (4.20)	**−8.6 (3.21)**

The late readiness potential mean amplitude was significantly (in bold) higher than the early readiness potential mean amplitude both in C3 and C4 channels.

### Affective Rating

After the EEG recording, ten out of the eleven participants evaluated how they had felt when interacting with each stimulus. ANOVA analysis revealed a main effect [F (2, 20) = 99.92, p<0.01] for valence. Post-hoc analysis revealed that the unpleasant stimuli (mean 2.9±0.99) scored significantly lower (p<0.01) than the neutral (4.9±0.29) and pleasant stimuli (7.1±0.69) and the neutral stimuli scored significantly lower than the pleasant stimuli (p<0.01). There was also a main effect for arousal [F (2, 20) = 26.00, p<0.01]. Fisher’s post-hoc test revealed that both the unpleasant (3.7±1.67) and the pleasant stimuli (4.4±1.22) scored similarly (p = 0.15) in arousal with both categories having significantly higher arousal scores (p<0.01) than that of the neutral stimuli (1.1±0.18).

## Discussion

The main purpose of this study was to investigate if the emotional valence of the stimulus with which one is about to grasp is taken into account during the motor planning of that action. As we hypothesized, compared to neutral stimuli, grasping pleasant stimuli was preceded by a reduced readiness potential whereas grasping unpleasant stimuli was preceded by an enhanced readiness potential.

Lower readiness potential amplitudes preceded the task of grasping pleasant stimuli and bringing them close to the chest. Additionally, participants rated the interaction with pleasant stimuli as more agreeable than with other categories. The amplitude of the readiness potential has been described as being inversely proportional to the easiness to perform a given movement [39]. Furthermore, faster reaction times are found after the viewing of pleasant as compared to neutral pictures [41]. Thus, lower readiness potential amplitudes might be the result of the congruence between the act of grasping and bringing stimuli toward the body and their pleasantness. Sets of approach-like movements such as holding, grasping and bringing objects toward the body are magnified in the primate frontal areas and heavily biased toward the central region of space in front of the chest [9], [42]. Besides the congruency between the nature of the upcoming action and the stimuli valence, the recruitment of approach-like sensorimotor repertoires, similar to those described in monkeys [9], might have led to lower readiness potential amplitudes.

Conversely, higher readiness potential amplitudes were found preceding the task of grasping unpleasant stimuli and bringing them close to the chest. Additionally, participants rated the interactions with unpleasant stimuli as more disagreeable than with other categories. Higher readiness potential amplitudes might result from the effort of grasping and bringing unpleasant stimuli close to the body. Experimental evidence suggests that the readiness potential magnitude preceding a self-paced movement is proportional to the degree of effort associated with its execution [43] and to the level of resource mobilization [38]. Neuroimaging studies showed increased activity in motor related areas when a simple reaction time task was performed after viewing unpleasant as compared to neutral pictures [19]. Furthermore, greater readiness potential amplitudes were detected preceding a button press movement performed after viewing unpleasant compared to neutral pictures [44]. Together with our findings, these results could be interpreted as a consequence of a discrepancy between the required action and an avoidance-like response evoked by the emotional context. In another line of evidence, microstimulation of the monkey pre-motor cortex leads to withdrawal movements of eye, lip and arm [9], [42]. Therefore, higher readiness potential amplitudes might result from the need to overpass preset networks to comply with the instructions to perform the task.

Another interesting result of this study was that the early and late readiness potential components were equally modulated by valence. The early readiness potential corresponds to a more general preparation for the forthcoming movement, with symmetrical distribution over the scalp and peak amplitude over midline fronto-central sites, mostly relative to the activity of the supplementary motor area. On the other hand, the late readiness potential, or negative slope, refers to a predominant activity over the hemisphere contralateral to the moving limb, and corresponds to the recruitment of primary motor (M1) and pre-motor cortices [34]. These two segments were analyzed separately to guarantee that any putative differential emotional modulation upon motor preparation would be captured. Our findings show that valence effects are present on both readiness potential segments, indicating that these effects span throughout motor preparation. Finally, the readiness potential measures did not reveal any valence-based laterality effect, although approach tendencies were found to activate the left prefrontal cortex whereas withdrawal tendencies, the right prefrontal cortex [45].

Previous studies investigated the influence of emotional picture viewing on corticospinal excitability elicited by transcranial magnetic stimulation [46]–[52]. Departing from these studies, where emotional stimuli were depicted as pictures and motor response was inexistent or unrelated to the emotional context, here the participants were asked to interact with three dimensional stimuli accommodated inside transparent cylinders. The use of such stimuli and a goal-directed action allowed a more realistic and evocative experimental setting. Thus, we show for the first time that the valence of the stimuli to which the action is directed affects the sensorimotor cortex activity preceding its grasping.

Emotional and neutral stimuli were carefully selected from a larger set so that pleasant and unpleasant categories would carry opposite valence and similar arousal. All stimuli were presented in identical transparent cylinders to prompt a similar grip. Participants were asked to execute the same movements throughout the experimental session. Additional care was taken to ensure similar weights among stimuli. These cautions are important to avoid biases effects due to different motor demands that might arise from distinct levels of force production or task complexity.

One could suppose that the simple viewing of the objects might had promoted affordance effects, *i.e.*, motoric activations due to the sight of graspable object [53], [54]. Affordance was recently described as taking place at about 250 to 300 ms after graspable objects’ presentation [55], [56].This interval is far before our window of analysis, which started at approximately 1500 ms relative to the movement onset. Moreover, our design was conceived in a way that the participant had to plan to grasp the transparent cylinder and not the object inside it. Additionally, when subjects were asked to judge the type of grasp evoked by the very same stimuli pictures, no significant difference among the three valence categories was found. Therefore the differences in readiness potential might not be due to any putative affordance effect of the objects inside the cylinders but to their emotional valence.

Skilled motor behavior relies on internal models that take into account the current state of the body, the external objects and the motor command to estimate the action outcomes beforehand [2], [57]–[59]. In this context, value needs to be predicted before any action is taken [3], [10]. As a matter of fact, value coding is considered as clearly behaviorally relevant [7], [10], [13]. For instance, it was shown that saccade peak speeds tend to be higher and less variable when they are done to rewarded target locations [6]. Besides, contemporary theories of emotion are based on the belief that, in order to survive, animals should be capable of identifying either threat signals that allow them to avoid risks to their body envelope or safety signals that allow them a sense of protection [12], [45]. In such perspective, one might benefit of predicting the objects’ valence with which one is about to interact. Herein, the readiness potential, an index of motor preparation, was shown to take into account the valence of to-be grasped stimuli. Therefore, we propose that the internal models of action might include valence predictions of to-be-grasped stimuli to estimate appropriate motor outcomes.

In conclusion, the present results show that emotion modulates motor preparation in such a way that movements directed to pleasant stimuli seems to be pre-set and easier to recruit than those directed to unpleasant stimuli. Thus, one could speculate that patients with motor impairment would improve their performance during a rehabilitation program if they were encouraged to interact with valence laden stimuli.

## Materials and Methods

### Ethics Statement

The study was approved by the Ethics Review Board of the University Hospital Clementino Fraga Filho from Federal University of Rio de Janeiro, number 004/09 and was carried out according to the Declaration of Helsinki. All participants provided informed consent before assessment.

### Stimuli Selection

To select thirty-nine stimuli (thirteen pleasant, thirteen unpleasant and thirteen neutral) to be employed in the experimental session a group of twenty-seven healthy male students (18–33 years of age) classified a set of sixty different items presented in transparent cylinders (height 9.7 cm and radius: 3.5 cm) sealed with a plastic film. A single gender sample was chosen because emotionally laden stimuli selection is often gender specific [60], [61].

Each stimulus was presented for six seconds to be appraised. After this period, a buzzer signaled that the participant should grasp the cylinder with his left hand, bring it close to the chest and replace it on the table. The participant was then given ten seconds to estimate how he felt about interacting with the stimuli. This evaluation was completed using the paper and pencil version of the Self-Assessment Manikin (SAM) [62]. Participants classified their interaction with each stimulus in the valence and arousal dimensions. The scale of the hedonic valence dimension is composed of pictorial drawings of manikins with expressions ranging from “smiling-happy” to “frowning-unhappy”. For the arousal dimension, the expressions of the manikins range from an excited, wide-eyed figure to a relaxed, sleepy figure. For analytic purposes, the ratings in the hedonic valence dimension were converted to numbers ranging from one (extremely unpleasant) to nine (extremely pleasant) with five representing neutrality. The ratings in the emotional arousal dimension were converted to numbers ranging from one (low arousal) to nine (high arousal).

Average ratings in valence and arousal dimensions were computed for each stimulus. Selection criteria for the thirteen representative stimuli of each category were: (i) neutral: valence ratings around “5” and arousal ratings around “1”; (ii) pleasant: valence ratings around “7” and arousal ratings around “5”; (iii) unpleasant: valence ratings around “3” and arousal ratings around “5”.

Although the objects were placed inside transparent cylinders to favor a similar grip, one could wonder if perceptual features of the object themselves would influence electrophysiological results. To test if the type of grasp each object affords was balanced among the three categories, we did a new experiment where a picture of each stimulus (objects inside the cylinder) was presented. Seventeen new participants were invited to indicate the type of grasp they would employ to interact with the objects in a forced choice task, “Precision grip” (characterized by opposition of the thumb to one or more of the other fingers) or “Power grip” (in which the fingers are flexed to form a clamp against the palm), [63]. One way ANOVA analyses showed no significant difference for the type of grasp among the three valence categories [F (2, 36) = 0.91, p = 0.41].

### Participants

A separate sample of seventeen right-handed male students from the Federal University of Rio de Janeiro (Brazil) aged twenty-one to thirty-six years (27.7±4.12 years) with no reported neurological or neuropsychiatric diseases participated in the electroencephalographic recording experiment. No participants reported taking any medication. Handedness was assessed by the Edinburgh inventory [64].

### Electrophysiological Recordings

#### Electromyography

Surface electromyographic activity (EMG) was recorded from the *extensor carpi radialis* muscle of the left arm as a marker of the initiation of movement. Two Ag/AgCl electrodes (diameter: 8 mm; inter-electrode distance: 2 cm) connected to the MP150 amplifier (BIOPAC Systems Inc.) were used. A reference electrode was fixed on the left lateral epicondylus. The EMG signal was acquired at a sampling rate of 1000 Hz and analogically filtered (band-pass: 10–350 Hz).

#### Electroencephalography

Electroencephalographic (EEG) signal was recorded from twenty gold electrodes (EMSAMED, Rio de Janeiro, BRAZIL) placed according to the international 10–20 system. Linked mastoids were used as references. The electrodes were fixed with a conductive gel. Impedances were kept below 5 kΩ. The EEG signal was sampled at 600 Hz and band-pass filtered between 0.1 and 35 Hz. As the readiness potential is more exuberant in the sensorimotor regions, a region-of-interest analysis was performed in Cz, C3 and C4 [26].

### Procedure

**Figure 2:**
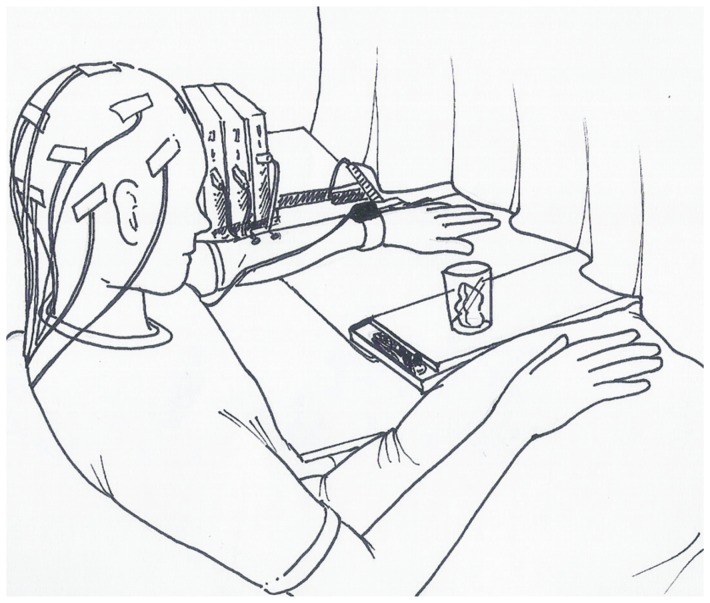
The participant sat with his left arm placed over a table. The right arm rested over a pillow for the duration of the session. An experimenter sat behind a black curtain and presented each cylinder fitted in a socket. Upon presentation, participants were instructed to wait, grasp the cylinder with the left hand and bring it close to the chest. Subjects were then asked to put the cylinder down on the tray socket and return the hand to the initial position. EEG and EMG signals were recorded throughout the experiment.

Participants were tested in a sound-attenuated room under dim ambient light. They were asked to sit at a comfortable chair with their left arm placed over a table. The right arm rested on a pillow all over the session. The cylinders were presented, one at a time, by an experimenter seated behind a black curtain (Figure 2). Each cylinder was presented in a fitted socket. A training session ensured that participants waited at least three seconds to initiate the movement upon stimulus presentation. The participants were instructed to grasp the cylinder with the left hand and bring it close to the chest. Then, they were asked to put the cylinder down on the tray socket and return the hand to the initial position. As the participant was instructed to decide when to begin the movement, the duration of stimuli presentation was not fixed. After the participant replaced his hand on the table, the experimenter changed the stimulus by another. The elbow remained positioned over the table during the entire movement. Participants were asked to perform the task with their left hands because a stronger readiness potential negativity was reported for movements with the non-dominant left hand [65].

In the training session nine stimuli (three of each category), not employed in the experimental session, were used. Feedback (“too fast”/“too slow”) about how long to wait until starting the movement was given by the experimenter after each trial.

The experiment was completed in four blocks, with three min rest intervals between each of them. In each block, the thirty-nine pre-selected stimuli (thirteen for each category) were displayed in a pseudo-randomized sequence with no more than three repetitions of the same category. To ensure attention engagement, the participants were instructed to carefully observe each stimulus to identify it afterwards. At the end of the fourth block, the electrodes were removed and the evaluation session began. The stimuli were presented again, one at a time, in the same sequence as in the previous block. The total duration of the experiment was approximately one hour and half.

### Affective Rating

The interaction with the thirty-nine stimuli composing the three experimental categories presented during electrophysiological recording was evaluated at the end of the experiment. The aim of this was to evaluate if the participants that performed EEG recording rate the interaction with unpleasant, pleasant and neutral stimuli as such. The participant was seated in a chair facing a table on which the stimuli were presented, one at a time, by an experimenter seated behind a black curtain. Each stimulus was presented. After six seconds of stimulus exposure, a buzzer signaled that the participant had ten seconds to rate how he had felt when interacting with that stimulus. This evaluation was performed in the valence and arousal dimensions using the pen and pencil version of the Self-Assessment Manikin (SAM) as in stimuli selection session, see above [62].

### Data Analyses

The EMG signal was rectified and digitally filtered (low pass: 10 Hz). Movement onset was attributed to the point in time where the EMG signal obtained 100 ms after stimulus presentation increased more than three standard deviations from resting activity. Furthermore, EMG activity should remain at or above this value for at least 500 ms. This automatic estimation was also verified by visual inspection.

The EEG signal was digitally filtered off-line with a Butterworth second order filter (low pass: 10 Hz). Off-line analyses used MATLAB software (Math works, USA). Epochs in which the EEG exceeded ±100 µV were eliminated from further analysis, thus excluding eye blink artifacts. Two participants were excluded from the analyses because more than half of their trials were eliminated. For the remaining participants, less than 27% of the trials were eliminated on this criterion. The percentage of rejection per channel was not statistically different among the categories.

The EEG analysis was performed within 2600 ms prior to the onset of the EMG burst. The baseline was set at 2600–2500 ms. Windows of interest relative to movement onset were then set at 1500–500 ms (early readiness potential) and 500–100 ms (late readiness potential) [34]. These EEG segments were grouped and averaged according to the stimuli category (pleasant, unpleasant and neutral) resulting in readiness potentials per participant per category. Mean amplitude values were calculated from early and late readiness potential slope periods. The data from three participants not showing a readiness potential in the neutral category were not further analyzed [31]. One participant presenting readiness potential amplitudes higher than three standard deviations above the group average for all categories was also excluded.

### Statistics

All analyses presented here were performed with data from the eleven remaining participants.

To appraise significant differences between the mean values of readiness potential amplitude, statistical analysis was performed using an ANOVA with repeated measures with the following factors: CATEGORY (pleasant, unpleasant and neutral), CHANNEL (C3, C4 and Cz) and WAVE SEGMENT (Early readiness potential and Late readiness potential). Data sphericity was verified prior to performing the statistical analysis (Mauchly’s test, P>0.05). ANOVA with repeated measures were also performed separately for the valence and arousal scores of stimuli interaction rating. In all cases, Fisher LSD post-hoc analysis was employed when significance was attained and the alpha level was set at 0.05. An one-way ANOVA was applied to search for differences in the type of grasp each object affords across categories.
